# Obituary

**Published:** 1901-06

**Authors:** 


### Obituary.

James Wilding, whose death took place a short time ago at his house in Fremantle Road, Bristol, was born at Liverpool in June, 1860, and was consequently in his 41st year. He received his medical education at the Westminster Hospital and Newcastle-on-Tyne. He took the degree of M.B. Durham in 1886 and the M.D. two years later; he had taken the diploma of L.S.A. in 1884, and those of M.R.C.S., L.R.C.P., in 1885. After two years' service at sea on board the ss. *Lord Gough* he settled in Bristol, and was appointed Surgeon to the Foresters' Medical Institution, which post he held for thirteen years till his death. Dr. Wilding contributed several papers to the medical journals, but the calls of a large practice prevented his having much time for such work. He was a very able practitioner, and enjoyed the esteem and confidence of his patients in a very marked degree, as indeed he well deserved to do, for he spared no pains in the care of his patients, and was unwearied in his devotion to all of them—to the poorest as well as to those who were better off. His loss will be deeply felt by a large circle of friends and patients who held him in high esteem. Seven years ago he suffered from an attack of acute rheumatism which damaged his heart, and on a second attack supervening complicated with pericarditis and pneumonia he passed away after a fortnight's illness. Only recently he had been appointed on a Sub-Committee of the Bath and Bristol Branch of the British Medical Association to consider the question of wage limit, a matter on which he was well qualified to speak, but his illness and death prevented the Committee from availing themselves of his assistance. He leaves a widow, but their only child died a few years ago.

Mr. Thomas Coomber, F.I.C., F.C.S., who died at his residence, 52 Clarendon Road, on May 21st, 1901, had been connected with the Bristol Medical School and University College as Lecturer on Chemistry for a period of nearly thirty years, as he was elected in 1868, and resigned his office in 1896. He was a very successful teacher in the Bristol Trade and Mining School, and when he resigned the Headmastership of the Merchant Venturers' School, about ten years ago, a handsome presentation of silver was made to him. His colleagues in the medical department were grateful for the energetic and useful part which he took in the early foundation of the University College in Bristol.

